# Adult Users of the Oticon Medical Neuro Cochlear Implant System Benefit from Beamforming in the High Frequencies

**DOI:** 10.3390/audiolres11020016

**Published:** 2021-04-16

**Authors:** Bianca Bastos Cordeiro, Marcos Roberto Banhara, Carlos Maurício Cardeal Mendes, Fabiana Danieli, Ariane Laplante-Lévesque, Chadlia Karoui, Michel Hoen, Marine Ardoint, Fanny Gauvrit, Romane Demullier, Christophe Vincent

**Affiliations:** 1 Institute of Health Sciences, Federal University of Bahia, Salvador 40110-100, Brazil; biancabastosfono@gmail.com (B.B.C.); mcardeal@ufba.br (C.M.C.M.); 2 Department of Life Sciences, Bahia State University, Salvador 41150-000, Brazil; marcos.banhara@irmadulce.org.br; 3 Oticon Medical, São Paulo 04360-001, Brazil; fadi@oticonmedical.com; 4 Oticon Medical, 2765 Smørum, Denmark; 5 Department of Behavioural Sciences and Learning, Linköping University, 581 83 Linköping, Sweden; 6 Oticon Medical, 06220 Vallauris, France; chok@oticonmedical.com (C.K.); mhoe@oticonmedical.com (M.H.); mard@oticonmedical.com (M.A.); 7 French National Institute of Health and Medical Research, 59016 Lille, France; fanny.gauvrit@chru-lille.fr (F.G.); romane.demullier@gmail.com (R.D.); christophe.vincent@chru-lille.fr (C.V.); 8 Department of Otology and Neuro-Otology, Lille University Hospital, 59000 Lille, France

**Keywords:** cochlear implants, directional microphones, beamforming, speech identification in quiet and in noise

## Abstract

The Oticon Medical Neuro cochlear implant system includes the modes Opti Omni and Speech Omni, the latter providing beamforming (i.e., directional selectivity) in the high frequencies. Two studies compared sentence identification scores of adult cochlear implant users with Opti Omni and Speech Omni. In Study 1, a double-blind longitudinal crossover study, 12 new users trialed Opti Omni or Speech Omni (random allocation) for three months, and their sentence identification in quiet and noise (+10 dB signal-to-noise ratio) with the trialed mode were measured. The same procedure was repeated for the second mode. In Study 2, a single-blind study, 11 experienced users performed a speech identification task in quiet and at relative signal-to-noise ratios ranging from −3 to +18 dB with Opti Omni and Speech Omni. The Study 1 scores in quiet and in noise were significantly better with Speech Omni than with Opti Omni. Study 2 scores were significantly better with Speech Omni than with Opti Omni at +6 and +9 dB signal-to-noise ratios. Beamforming in the high frequencies, as implemented in Speech Omni, leads to improved speech identification in medium levels of background noise, where cochlear implant users spend most of their day.

## 1. Introduction

Persons with hearing impairments, including cochlear implant users, have difficulty following conversations when the signal is acoustically degraded, for example due to noise or reverberation [1]. Analyses of several thousands of cochlear implant (CI) datalogs show that CI users spend twice as much time listening to speech in noise than speech in quiet [2], and this pattern holds for all age groups.

Listening is cognitively taxing for CI users, as electroencephalography and pupillometry studies show [3,4,5]. This results in fewer cognitive resources remaining for other tasks, such as information processing and storage, and, over time, may lead to listening fatigue [6]. However, several signal processing features can improve CI listening.

### 1.1. Signal Processing to Improve Cochlear Implant Listening

Directional microphones are a form of noise reduction based on acoustic beamforming with multi-microphone algorithms that exploit the spatial information of the sound sources [7]. With directional microphones, the input collected by several microphones is combined to result in reduced sensitivity for some angles of arrival (azimuth), typically located at the sides and the rear of the listener. Directional microphones improve the SNR and therefore listening performance of CI users [8,9]. Directional microphones can have fixed or adaptive patterns of sensitivity to sounds from different angles of arrival [7]. By steering their sensitivity away from the dominant noise sources, adaptive directional microphones provide superior benefits over fixed directional microphones in CI users [10]. As directional microphones achieve spatial selectivity based on differences in angles of arrival, their benefits are largest when the signal is coming from the front, when signal and noise come from different directions (“spatially separated”), when there is only one signal source and one noise source, and when reverberation is limited.

### 1.2. Neuro CI System by Oticon Medical

The Neuro CI system by Oticon Medical has been available for children and adults since 2015 [11,12]. The Neuro Zti implant has a 20-channel electrode array. The Neuro CI system sound processing is identical in its two generations of sound processors, Neuro One and Neuro 2. It uses Coordinated Adaptive Processing [13], which includes Free Focus (automatic adaptive multiband directionality), Voice Guard (output compression), Wind Noise (dual-microphone wind noise reduction algorithm), and Voice Track (single-channel noise reduction). Free Focus benefits from a monaural adaptive beamformer. Two microphones placed along the front–back axis of the behind-the-ear sound processor capture the incoming sound signal. The input/output characteristics of the microphones are linear, and the total harmonic distortion is below 1% for sound inputs from 10 to 113 dB SPL. The frequency response of the microphones is known from 1.1 to 10 kHz. Frequencies higher than 8 kHz are not processed for stimulation. During manufacturing, the two microphones are paired (i.e., their sensitivity levels are matched within ± 1 dB) to ensure directionality benefits. The signal that the microphones capture is divided into four frequency bands.

**Figure 1:**
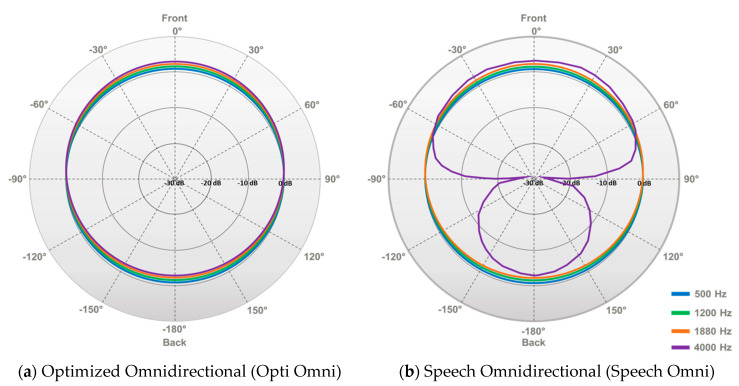
Polar patterns of the two modes (**a**) Optimized Omnidirectional (Opti Omni) and (**b**) Speech Omnidirectional (Speech Omni). Note that (0 azimuth is at the top and that the two plots are normalized re. 90 azimuth. (**a**) In the Opti Omni mode, sensitivity to all angles of arrival is equal, except that sounds originating from the back, regardless of frequency, are slightly attenuated compared to sounds originating from the front (mild front advantage). (**b**) In the Speech Omni mode, the microphone response is the same as Opti Omni in the low frequencies. In the high frequencies (cut-off frequency of 1.88 kHz), the directional microphones attenuate sounds originating from the back.

In the Genie Medical CI software, the professional selects one of two Surround modes: Optimized Omnidirectional (Opti Omni) or Speech Omnidirectional (Speech Omni). These two modes vary in how the directional microphones act and, therefore, their spatial selectivity. In the Opti Omni mode, sensitivity to all angles of arrival is equal, except that sounds originating from the back, regardless of frequency, are slightly attenuated compared to sounds originating from the front (mild front advantage). In the Speech Omni mode, the microphone response is the same as Opti Omni in the low frequencies. In the high frequencies (cut-off frequency of 1.88 kHz), the directional microphones attenuate sounds originating from the back. This is intended to provide help in moderate levels of background noise. Speech Omni is therefore more directional than Opti Omni (see Figure 1). As CI users are mostly in situations with some noise, Speech Omni is set as the default Surround mode. The professional can change the Surround mode to Opti Omni in Genie Medical CI.

### 1.3. Previous Studies Comparing Opti Omni and Speech Omni in Hearing Aid and Cochlear Implant Users

In an earlier study, 48 Oticon hearing aid users with mild to moderately severe symmetrical sensorineural hearing impairment repeated HINT sentences presented from the front and unmodulated noise presented from azimuth +45, +135, −135, and −135 degrees at a level of 65 dB SPL [14]. In a crossover trial, the participants used Opti Omni or Speech Omni for 10–14 days, completed the HINT test with that first mode, used the mode not yet trialed for 10–14 days, and completed the HINT test with that second mode. Participants could identify the target sentences at significantly poorer SNRs with Speech Omni than with Opti Omni (*p* = 0.045). However, they were equally likely to report a preference for either mode. In another study, 35 Neuro CI system users received two sound processor maps where the only difference between the two maps was whether Opti Omni or Speech Omni was activated [15]. The participants were asked to report whether they preferred using Opti Omni or Speech Omni or whether they had no preference in 10 different listening situations. Participant preference for Speech Omni was significantly above the chance level. However, the speech identification performance of CI users with Opti Omni and Speech Omni have yet to be compared.

### 1.4. Measuring Directional Microphone Benefit in Cochlear Implant Users

Directional microphones have been commercially available in CIs for over 15 years. Several studies have documented directional microphone benefits in terms of the speech identification performance in CI users, both after the provision of a habituation period, during which the user trialed the microphone mode at home before laboratory testing [16,17,18], or without such a habituation period [9,19,20,21,22,23,24,25]. Several studies have shown that, compared to an omnidirectional microphone, the directional microphone benefit for CI users in anechoic or low reverberation environments is around 3.5–6 dB [17,18,19,20,21,23,24,25]. Several types of signals and noise, SNRs, and angles of arrival have been used. For example, the noise can be stationary or fluctuating (e.g., babble noise). Noise sources can be single or multiple (diffuse), their location can be fixed or changing, and they can be co-located or spatially separated from the signal. Beyond the provision of a habituation period, only some studies randomized conditions during laboratory testing and blinded participants and experimenters to the mode tested.

### 1.5. Research Aim

The overall research aim was to measure and compare speech identification scores in quiet and in spatially separated noise with the Opti Omni and the Speech Omni modes in adult Oticon Medical Neuro CI system users. Two independent studies were carried out to measure the impact of these two modes on speech identification performance. The hypothesis was that Speech Omni, due to its increased spatial selectivity, i.e., sensitivity to sounds coming from the front, compared to Opti Omni, would lead to significantly better speech identification performance in noise in both studies.

## 2. Materials and Methods

Both studies compared Opti Omni and Speech Omni by selecting the relevant Free Focus mode in Genie Medical CI. Automatic switching between modes was deactivated and no other signal processing contrasts were introduced.

The two studies had different objectives and therefore were conducted using different methodologies and on samples with different characteristics. Study 1 investigated differences in sentence identification scores with Opti Omni and Speech Omni at a fixed SNR in a typical group of relatively new CI users, after a period of habituation to the two modes. Study 1 used a sentence identification test with a fixed SNR (+10 dB). Therefore, consecutive CI candidates were enrolled in Study 1. Study 1 used a double-blind longitudinal crossover design to evaluate sentence identification in quiet and in noise with fixed SNR in the two modes Opti Omni and Speech Omni. The CI listeners had a three-month habituation period in each of the modes prior to testing.

Study 2 investigated the SNRs at which any difference in sentence identification scores between Opti Omni and Speech Omni would be observed. Study 2 used a sentence identification test with adaptive SNRs (ranging from −3 to +18 dB). Therefore, CI users with ≥6 months of experience and who scored ≥40% on a test of monosyllabic speech identification were enrolled in Study 2. Study 2 explored speech identification performance in the two modes, Opti Omni and Speech Omni, with adaptive multi-talker babble noise presented from multiple locations around the listener.

### 2.1. Study 1: Speech Identification with Opti Omni and Speech Omni after Habituation

#### 2.1.1. Summary of Methods

This longitudinal crossover study was conducted at Santo Antônio Hospital, Charitable Works Foundation of Sister Dulce in Salvador, Brazil. Twelve new adult Neuro CI system users trialed the Free Focus mode Opti Omni or Speech Omni (random allocation) for three months, and their sentence identification in quiet and in noise (+10 dB SNR) with the trialed directionality mode was measured. The users then trialed the mode they had not trialed yet for three months and their sentence identification scores in quiet and in noise were measured with this second mode.

#### 2.1.2. Participants

The participants were 12 new adult Neuro CI system users. Potential participants were identified from the Santo Antônio Hospital patient database of CI candidates and were invited to take part in this study. They met the standard CI candidacy criteria: they had a bilateral severe and/or profound sensorineural hearing impairment and they derived limited benefits from hearing aids (sentence identification < 50% in the best-aided ear). In addition, inclusion criteria were the following: aged ≥18 years with a post-lingual hearing impairment, a candidate for their first CI surgery, and fluent in Brazilian Portuguese.

#### 2.1.3. Procedure

After recruitment, all participants underwent unilateral cochlear implantation and received a Neuro Zti implant and a Neuro One or Neuro 2 sound processor. They attended three study sessions at Santo Antônio Hospital. They used Opti Omni for the first three months after CI activation. Their sentence identification scores were then measured and have been published elsewhere [26]. Then, participants were randomly assigned in a 1:1 fashion to use either Opti Omni or Speech Omni for three months. Block randomization was used: the first 6 participants recruited trialed Speech Omni first, and the last 6 participants recruited trialed Opti Omni first. At 6 months, the participants completed sentence identification tests in the mode they had trialed first. Following a crossover design, the directional mode was changed to the one not used during the first evaluation. Participants used the new mode for three months and returned at 9 months for the second sentence identification test. Participants were blinded to the specifics aims of the study and to which modes they were trialing.

The open-set Lists of Phrases in Portuguese (LPP) sentence identification test [27] was used. The test includes seven lists of ten sentences each. The sentences are phonetically balanced [28] and the lists are equivalent [29]. Each sentence contains 4–7 words that are weighted differently [30]. Correctly repeated words with lexical or semantic meaning, such as nouns, adjectives, verbs, adverbs, and numerals score 2 points. Correctly repeated words with grammatical meaning, such as articles, prepositions, conjunctions, pronouns, and interjections score 1 point. As the sentences have different lengths, total scores are multiplied by a correction value, so they are expressed in percentages, as previously published [30]. Examples of sentences include “O avião já está atrasado” (the plane is already late) and “O preço da roupa não subiu” (the price of clothing has not gone up). The audiologists scoring the sentences were blinded to which mode each participant was trialing.

Participants were seated in a soundproof booth. Participants who used a contralateral hearing aid removed it for the testing. All participants were therefore tested whilst listening through one Neuro CI system. The sentences were presented at 65 dB SPL from a loudspeaker positioned in front of the participants (azimuth 0 degrees), one meter away. Speech-shaped stationary noise was presented at 55 dB SPL from a loudspeaker positioned behind the participants (azimuth 180 degrees), one meter away. The SNR was fixed at +10 dB. The participants were presented with one list of ten sentences for each condition.

### 2.2. Study 2: Speech Identification with Opti Omni and Speech Omni at Various Signal-to-Noise Ratios

#### 2.2.1. Summary of Methods

This study was conducted at the Lille University Hospital in Lille, France. The sentence identification performance of 11 experienced adult Neuro CI system users were compared in quiet and at relative SNRs ranging from −3 to 18 dB when using Opti Omni and Speech Omni. The focus of this study was on the benefits of Speech Omni over Opti Omni at different SNRs.

#### 2.2.2. Participants

Participants were 11 experienced adult Neuro CI system users. Potential participants were identified from the Lille University Hospital patient database and were invited to take part in this study if they had received a Neuro Zti implant and used a Neuro 2 sound processor. Further inclusion criteria were the following: aged ≥18 years and <85 years, post-lingual hearing impairment, sound processor activated for >6 months, monosyllabic speech identification with CI ≥ 40%, and fluent in French. As this study also recorded pupillometry (results not reported here), they also had no ophthalmological or motor disorders resulting in head tremors.

#### 2.2.3. Procedure

The tests occurred in one session at the Lille University Hospital. One experimental Neuro 2 sound processor was used for all participants. The sound processor map of each patient was imported into the experimental sound processor and then copied into two programs, with the only difference between the two programs being the Surround mode, Opti Omni or Speech Omni. Before the study, ten patients had a single program (Tri-mode with Speech Omni for nine patients and Tri-mode with Opti Omni for one patient) and one patient had three programs: Opti Omni, Speech Omni, and Full Directionality. Participants were blinded to the specific aims of the study and to which modes they were trialing.

The open-set Vocale Rapide dans le Bruit (VRB) sentence identification test [31] was used. The test includes 15 lists of nine sentences each. The sentences are phonetically balanced [31]. Each sentence contains 4–10 words; of these, three are identified as keywords and scored. Examples of sentences include “Il pleut depuis hier matin” (it is raining since yesterday morning) and “Il fait trop chaud pour sortir faire des courses” (it is too warm to go out shopping). Due to limited personnel, the person scoring the sentences was not blinded to which mode each participant was trialing.

The VRB is an adaptive sentence-based test like the QuickSIN [31]. Sentences are presented at 65 dB SPL. The first sentence is presented in quiet and the following eight sentences are presented in noise whose level increases in steps of 3 dB. VRB test results are reported as SNR levels relative to a mean speech reception threshold (SRT), i.e., the SNR where people with normal hearing acuity achieve a score of 50%, derived from a mean psychometric function obtained in adults with normal hearing acuity. VRB sentences were adjusted so that the level of 0 dB SNR is the mean SRT measured in people with normal hearing acuity [31]. Sentences are presented at relative SNRs from this point, ranging from −3 to 18 dB. Scoring is based on the three keywords of the eight sentences presented in noise. The Spearman–Kärber equation is used to calculate the 50% threshold on the psychometric function, i.e., the listener’s SRT, relative to people with normal hearing acuity. Therefore, in the VRB test, the SRT results represent a deviation from normal hearing scores, or “SNR loss” [31].

Participants were seated in a soundproof booth. Ten of the 11 participants were implanted unilaterally. One patient had a Neuro Zti implant in one ear and an implant from a previous generation in the other ear: the sound processor of the other ear was removed for the testing. Participants who used a contralateral hearing aid removed it for the testing. All participants were therefore tested whilst listening through one Neuro CI system. Five loudspeakers were placed 1.5 m from the participant (azimuth 0, +60, +120, −120, and −60 degrees). The sentences were presented at 65 dB SPL from a loudspeaker positioned in front of the participants (azimuth 0 degrees). Four-talker babble (i.e., fluctuating masker) noise was presented from the front and sides (azimuth 0, +60, +120, −120, and −60 degrees). For every sentence, the same 8 s four-talker babble excerpt was presented from all five loudspeakers in a synchronous fashion. The researcher who scored the sentences was not blinded to the mode tested. The protocol indicated that participants should complete up to 4 lists of sentences with each mode. During the testing, it became apparent that completing 4 lists was taxing for some participants. When participants showed signs of fatigue, they were tested with 2–3 lists for each mode. Averages across lists completed for each participant and each mode were used for the analyses.

Table 1 summarizes the methods and participants for the two studies.

### 2.3. Statistical Analyses

Analyses were conducted in RStudio Team (RStudio Inc., Boston, MA, USA) and in Statistica version 10 (StatSoft Inc., Tulsa, OK, USA).

For each study, a linear mixed-effects (LME) model with a random intercept for participants was fitted to the data (lmerTest package [32]). To further explore Study 2, a repeated-measures ANOVA as well as a t test comparing SRTs with Opti Omni and Speech Omni were conducted. Statistical significance was defined as *p* ≤ 0.05. There were no missing data to report.

## 3. Results

### 3.1. Study 1: Speech Identification with Opti Omni and Speech Omni after Habituation

**Figure 2:**
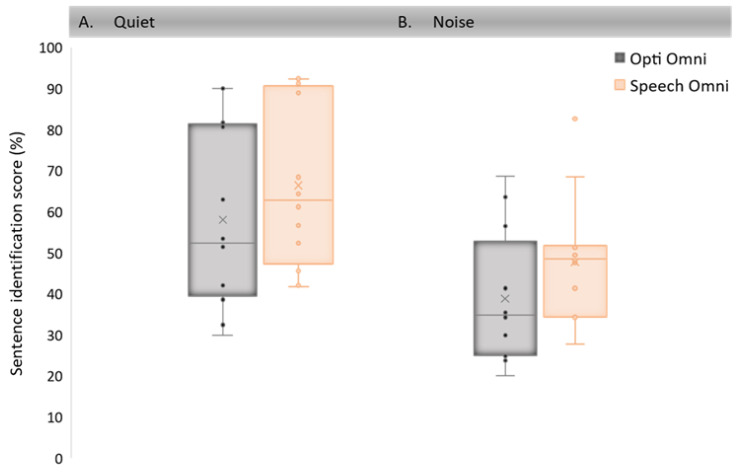
Boxplots of speech identification performance with the Speech Omni and the Opti Omni modes: (**A**) in quiet; (**B**) in noise for 12 CI Neuro users. Middle lines show medians; crosses refer to the means. The box ends show the first and third quartiles and the whiskers show 1.5 times the interquartile range below the first quartile and above the third quartile. Dots represent individual data. Outliers outside the boxplots are at least three times the interquartile range, below the first quartile or above the third quartile.

Sentence identification scores in quiet and in noise were obtained in 12 new CI users at 6 and 9 months post-activation, whether with the Opti Omni or with the Speech Omni modes according to the randomization procedure. Figure 2 illustrates the mean sentence identification scores of the 12 participants after three months of use of each mode. A linear mixed model was applied to the data with a random intercept for participants and defined fixed effects for the mode (Opti Omni vs Speech Omni), the test condition (quiet vs noise), the randomization sequencing (crossover design, 1 vs. 2) and the testing interval (i.e., testing interval after cochlear implantation). Overall, significant effects were found with the mode (Opti Omni vs. Speech Omni; F = −10.74, *p* = 0.002) and the test condition (quiet vs noise; F = 50.03, *p* < 0.001). The effects of the randomization sequencing and the testing interval were assessed and were not found to be significant.

Pairwise Tukey-adjusted comparisons show that speech identification scores were significantly higher with the Speech Omni mode than with the Opti Omni mode both in quiet (*p* = 0.001) and in noise (*p* = 0.001). Furthermore, speech identification scores were significantly higher in quiet than in noise for both the Opti Omni (*p* < 0.001) and the Speech Omni (*p* < 0.001) modes.

### 3.2. Study 2: Speech Identification with Opti Omni and Speech Omni at Various Signal-to-Noise Ratios

Speech identification scores in quiet and various SNRs were obtained in 11 experienced CI users with Opti Omni and Speech Omni in a single session at relative SNRs ranging from −3 dB to +18 and in quiet for each mode. As expected, scores were higher in quiet and at more positive SNRs. As in Study 1, a linear mixed-effect model with a random intercept for participants was fitted to the data. The analysis revealed an overall significant effect for both the test mode (Opti Omni vs Speech Omni; F = −7.98, *p* = 0.005) and the SNR condition (quiet vs adaptive SNRs; F = 96.96, *p* < 0.001).

Table 2 summarizes the post hoc pairwise analyses that revealed significant differences between speech identification scores in different listening conditions (i.e., quiet or different SNRs). No significant difference in performance could be seen within the −3 to +3 dB SNR range, between +3 and +6 dB SNR, and within the quiet condition and the +15–18 dB SNR range.

To further investigate the association between speech performance, the mode, and the different SNRs, a repeated-measures ANOVA was conducted, with mean sentence identification scores as the dependent variable for each participant at +3, +6, +9, +12, +15 and +18 dB SNR. To keep proper dataset dispersion, scores in quiet were not considered because they were close to 100% (ceiling effect). Similarly, scores at 0 dB SNR and −3 dB SNR were not considered because they were close to zero (floor effect). The analysis had two factors: the mode (two levels: Opti Omni vs Speech Omni) and SNRs (six levels: +3, +6, +9, +12, +15, and +18 dB SNR). The analysis shows a main effect of both factors, respectively for mode (F (1,10) = 9.61; *p* = 0.011) and SNR (F (5,50) = 41.34; *p* < 0.001), with a non-significant interaction. Multiple comparisons of the modes reported that for the six SNR conditions, the observed differences between the Opti Omni and the Speech Omni modes were significant at +6 and +9 dB SNR, with F = 2.09, *p* = 0.016 and F = 8.69, *p* = 0.014, respectively.

**Figure 3:**
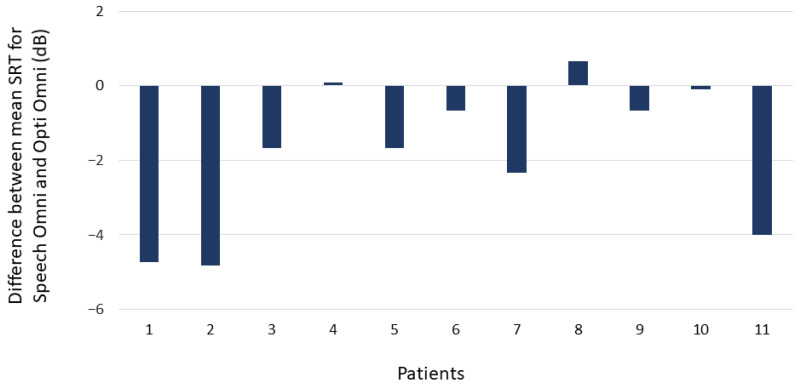
Difference between mean SRT (50% correct) for Opti Omni and Speech Omni for each of the 11 participants. A negative value indicates better scores with Speech Omni than with Opti Omni.

The mean SRT was 11.23 dB SNR (SD = 2.05) in the Opti Omni mode and 9.42 dB SNR (SD = 1.99) in the Speech Omni mode. In other words, Speech Omni shifted the point on the psychometric function where participants scored 50% by 1.81 dB. As the SRT scores followed a normal distribution (Shapiro–Wilk test W = 0.97, *p* = 0.71), a t test was conducted and showed that the difference in mean SRT was statistically significant (t = 11.94, *p* = 0.001). Figure 3 illustrates this difference for each the 11 individual participants. The SRTs were 3 to 5 dB better in four participants and 1 to 3 dB better in two participants with Speech Omni over Opti Omni. The SRTs were from −1 to 1 dB difference in five participants.

## 4. Discussion

Both studies demonstrated that Speech Omni significantly improves speech identification scores compared to Opti Omni. In Study 1, after three months of habituation, sentence identification scores in quiet and in noise (+10 dB SNR) were significantly better with Speech Omni than with Opti Omni in 12 new adult CI users. In Study 2, sentence identification scores were significantly better with Speech Omni than with Opti Omni at +6 and +9 dB SNRs, but not in the other tested conditions, in 11 experienced adult CI users. Speech Omni led to a 3–5 dB improvement in four participants’ SRTs and a 1–3 dB improvement in two participants’ SRTs. The two modes tested, Opti Omni and Speech Omni, are the Surround modes available in Oticon Medical CIs, and Study 2 showed a mean improvement of 1.81 dB in SRTs from Opti Omni to Speech Omni. The present study did not measure the benefit of the two directionality modes, Split Directionality and Full Directionality, modes similar to other commercially available directional microphones [17,18,19,20,21,23,24,25]. Taken together, the results show that Speech Omni improves speech identification both with a single noise source, presented at azimuth 180 degrees in Study 1, as well as with multiple noise sources, presented at azimuth 0, +60, +120, −120, and −60 degrees in Study 2. In Study 1, scores with Speech Omni were significantly better than scores with Opti Omni, both in quiet and in noise. The effect observed in quiet may be due to the increased front sensitivity for high frequencies, whilst the effect observed in noise may be due to a combination of the increased front sensitivity and the decreased back sensitivity for high frequencies (see Figure 1). The results also show that the Speech Omni benefits are measured after habituation (as in Study 1) but also without any habituation period (as in Study 2). Furthermore, Study 2 shows that Speech Omni leads to significantly better speech identification than Opti Omni in the middle of the psychometric function. Speech identification performance was characterized by floor effects at very low SNRs and ceiling effects at very high SNRs, both with Opti Omni and Speech Omni. The middle of the psychometric functions corresponds to medium levels of background noise (i.e., +6 and +9 dB SNR), where CI users spend most of their day [2]. In no condition tested was performance with Speech Omni significantly worse than with Opti Omni.

Although both studies report on the sentence identification performance of adult Neuro CI system users, the samples were different. Study 1 participants were tested after six and then nine months of CI experience, whilst Study 2 participants had mean CI experience of over three years. Study 1 prospectively followed up a group of CI recipients following surgery irrespective of their speech identification ability, whilst it was a criterion for inclusion that Study 2 participants scored ≥40% on monosyllabic word speech identification when presented at 65 dB SPL. This might explain why Study 2 participants reached better scores than Study 1 participants on their respective tests of open-set sentence identification. However, the test and their scoring method differed. Furthermore, the level and type of noise as well as speaker configuration were different between the two studies. All these factors have been reported to influence sentence identification scores [33]. For these reasons, it would be misleading to compare the scores of both studies.

### 4.1. Strengths and Limitations

Laboratory evaluations of speech identification such as the ones reported here aim to approximate real-life listening demands. Whilst recognizing that the nature of realistic test scenarios is not the same for two people, the two studies presented here considered aspects of speech materials, noise type and source(s), and SNRs that represent typical listening conditions for CI users. As highlighted recently, both noise fluctuations, from stationary noise to dynamic babble noise, and noise spatial configuration, from co-location to spatial separation, are important factors when measuring directional microphone benefit in CI users [34]. Taken together, the two studies explored different noise maskers and spatial configurations, adding to the robustness of the results observed. Blinding, which occurred in both studies, also adds to the robustness of the results observed.

When looking at each study in isolation, Study 1 participants benefited from a habituation period with a mode before the testing took place, and then crossing over to trial the other mode. All participants used Opti Omni for the first three months following activation before they were randomized to either a three-month habituation period with Opti Omni or Speech Omni. In total, all participants had six months of experience with Opti Omni and three months of experience with Speech Omni. This could have led to better speech identification performance with Opti Omni, but the contrary was observed. The Study 2 masker was the same four-talker babble excerpt presented from five loudspeakers in a synchronous fashion, which could have led to potential constructive and destructive interferences in the test environment. Presenting different maskers would reduce the likelihood of unwanted acoustic effects, as well as increase the ecological validity of the test setup.

To the authors knowledge, no other published reports of CI user performance on the two speech materials used, the Brazilian Portuguese-language LLP sentences and the French-language VRB sentences, are available. This limits the comparison of the present studies’ results with other results. More widely used speech materials such as AzBio and HINT are more suited to cross-study comparisons [35].

### 4.2. Clinical Implications

Speech Omni is the default Surround mode in Neuro 2 sound processor. The professional can change this to Opti Omni, but in light of new evidence showing that CI users spend most of their time in environments with some noise, together with the combined results of the two present studies, it appears that most adult CI users will benefit from a directional advantage in the high frequencies. Similarly, based on the results of Study 1, all Study 1 participants were fitted with Speech Omni as the default Surround mode after the conclusion of the study. However, further work should evaluate whether other population groups (e.g., pre-lingually deafened children) derive similar benefits before extending these recommendations beyond the population of the post-lingually deafened adults tested in the two present studies.

### 4.3. Future Research

Measures of listening effort could be used to investigate the effect of CI signal processing beyond speech identification performance, to assess the impact of an SNR improvement on the allocation of cognitive resources to listening versus other cognitive tasks. For example, pupillometry can be used for this purpose. Ecological momentary assessment could also be used to assess the benefits when faced with real-life listening demands, for example, through the CI user trialing and reporting on the benefits of different modes through an application.

## 5. Conclusions

This study shows significant benefits of beamforming in the high frequencies for adult CI users.

## Figures and Tables

**Table 1:** Summary of the methods and participants for the two studies.

	Study 1	Study 2
Center and Country	Santo Antônio Hospital, Charitable Works Foundation of Sister Dulce in Salvador, Brazil	Lille University Hospital in Lille, France
Participants	12 Neuro One sound processor users	11 Neuro 2 sound processor users
Sex	7 women, 5 men	5 women, 6 men
Age	Mean = 39.8 years	Mean = 55.2 years
	SD = 10.9 years	SD = 18.4 years
	Min = 21 years	Min = 19 years
	Max = 54 years	Max = 81 years
CI experience ^1^	Mean = 0.2 year	Mean = 3.7 years
	SD = 0.0 year	SD = 2.5 years
	Min = 0.2 years	Min = 1 year
	Max =0.2 years	Max = 18 years
Deafness duration	Mean = 11.9 years	Mean = 5.1 years
	SD = 9.8 years	SD = 5.2 years
	Min = 1 year	Min = 1 year
	Max = 30 years	Max = 19 years
Testing configuration	One Neuro cochlear implant system	
Stimuli type	Open-set sentences	
Stimuli language	Brazilian Portuguese LLP [30]	French VRB [31]
Speech stimuli presentation level	65 dB SPL	
Stimuli position	One loudspeaker: Azimuth 0 degrees	
Noise type	Speech-shaped noise i.e., stationary noise	Four-talker babble i.e., fluctuating masker
Noise presentation level(s)	55 dB SPL	47 to 68 dB SPL, increasing in 3 dB steps
Noise position	One loudspeaker: Azimuth 180 degrees	Five loudspeakers: Azimuth 0, +60, +120, −120, and −60 degrees
Signal-to-noise ratio	+10 dB	+18 to –3 dB, reducing in 3 dB steps

^1^ At the first of study visits for Study 1 participants; at the only study visit for Study 2 participants.

**Table 2:** Summary of post hoc *p*-values in the adaptive VRB test according to the different quiet and noise (relative dB SNRs) conditions.

	−3	0	3	6	9	12	15	18	Quiet
−3									
0	1.000								
3	0.996	0.099							
6	0.001	0.002	0.099						
9	<0.001	<0.001	<0.001	0.014					
12	<0.001	<0.001	<0.001	<0.001	0.005				
15	<0.001	<0.001	<0.001	<0.001	<0.001	1.000			
18	<0.001	<0.001	<0.001	<0.001	<0.001	0.478	0.973		
Quiet	<0.001	<0.001	<0.001	<0.001	<0.001	0.008	0.161	0.990	

## Data Availability

Not Applicable.
